# Berkshire Medical College Commencement

**Published:** 1864-12

**Authors:** 


### BERKSHIRE MEDICAL COLLEGE COMMENCEMENT.

The following are the names of the candidates for graduation, the subjects of their theses and their residences:

Daniel D. Gilbert, Boston, Exosmosis and Endmosis.
Charles L. Holt, Albany, Me., Dysmenorrhœa,
Marquis Hall, Bimfield, Pneumonia.
Wm. W. B. Green, Providence, R. I., Diagnosis.
W. H. H. Shepard, Warren, Gun Shot Wounds.
Henry J. Millard, Stamford, Vt., Phthisis Pulmonalis.
F. J. Swift, Wilmington, Vt., Duties of a Physician.
J. E. Norwood, Livingston, N. Y., Inflammation.
E. M. Whiten, Southport, Me., Diptheria.
Frank K. Paddock, Hamilton, N. Y., Venesection.
G. A. Wilder, Circleville, Ohio, Intermittent Fever.
Cyrus Allen, Palmyra, N. Y., Stricture of Urethra.
Henry Eastman, Pittsfield, Dysentery.
E. McCollom, Jr. Rochester, Vt., Retrospectus.
D. Sherwood Eckler, Athens, N. Y., Scarletina.
E. Newton Beale, Spencertown, N. Y., Inflammation.

Two prizes were awarded for the best specimens of practical anatomy; the first to F. J. Swift, of the graduating class; the second to M. S. Chamberlain, of Brimfield, under graduate.

The commencement prayer was offered by Rev. Dr. Todd, of the Board of Trustees. The annual address was given by Dr. O. S. Root, Secretary of the College Government. The subject was on the "Errors and Delusions of the Medical Profession," those which do not amount to professional heresy, or to quackery prepense. It was a sort of medical *concio ad clerum.* He prescribed the purgative to the physician—applied the knife to the surgeon—and we dare say the operation will be more beneficial to the patient than doses of honeyed flattery applied *ad nauseam*, or dissections of absent "irregular practitioners."

The diplomas were delivered to the graduates, with pertinent counsel and encouragement, by the venerable President and founder of the College, Hon. H. H. Childs, M. D., who, although exhibiting a frailer state of health than his friends could wish, displayed all the enthusiastic love of his earlier days for the Institution. [Com.

